# FH535 increases the radiosensitivity and reverses epithelial-to-mesenchymal transition of radioresistant esophageal cancer cell line KYSE-150R

**DOI:** 10.1186/s12967-015-0464-6

**Published:** 2015-03-31

**Authors:** Huafang Su, Xiance Jin, Xuebang Zhang, Lihao Zhao, Baochai Lin, Lili Li, Zhenghua Fei, Lanxiao Shen, Ya Fang, Huanle Pan, Congying Xie

**Affiliations:** Radiotherapy and Chemotherapy Deparment, the 1st Affiliated Hospital of Wenzhou Medical University, No.2 Fuxue Lane, 325000 Wenzhou, China

**Keywords:** Esophageal cancer, Epithelial-mesenchymal transition, FH535, Radioresistance, Wnt/β-catenin pathway

## Abstract

**Background:**

Acquired radioresistance has significantly compromised the efficacy of radiotherapy for esophageal cancer. The purpose of this study is to investigate the roles of epithelial-mesenchymal transition (EMT) and the Wnt/β-catenin signaling pathway in the acquirement of radioresistance during the radiation treatment of esophageal cancer.

**Methods:**

We previously established a radioresistant cell line (KYSE-150R) from the KYSE-150 cell line (a human cell line model for esophageal squamous cell carcinoma) with a gradient cumulative irradiation dose. In this study, the expression of EMT phenotypes and the Wnt/β-catenin signaling pathway proteins were examined by real-time PCR, western blot and immunofluorescence in the KYSE-150R cells. The KYSE-150R cells were then treated with a β-Catenin/Tcf inhibitor FH535. The expressions of nuclear and cytoplasmic β-catenin and EMT markers in KYSE-150R cells were assessed at both mRNA and protein level after FH535 treatment. The radiosensitization effect of FH535 on KYSE-150R was evaluated by CCK8 analysis and a colony forming assay. DNA repair capacities was detected by the neutral comet assays.

**Results:**

KYSE-150R cell line displayed obvious radiation resistance and had a stable genetic ability. EMT phenotype was presented in the KYSE-150R cells with decreased E-cadherin and increased snail and twist expressions. The up-regulated expressions of Wnt/β-catenin signaling pathway proteins (Wnt1, FZD1-4, GSK3β, CTNNB1 and Cyclin D1), the increased phosphorylation of GSK3β, and the decreased phosphorylation of β-catenin were observed in KYSE-150R cells compared with KYSE-150 cells, implicating the activation of the Wnt pathway in KYSE-150R cells. The expression of nuclear β-catenin and nuclear translocation of β-catenin from the cytoplasm was decreased after FH535 treatment. FH535 also reversed EMT phenotypes by increasing E-cadherin expression. The cell proliferation rates of KYSE-150R were dose-dependent and the radiation survival fraction was significantly decreased upon FH535 treatment. Neutral comet assays indicated that FH535 impairs DNA double stranded break repair in KYSE-150R cells.

**Conclusions:**

Acquisition of radioresistance and EMT in esophageal cancer cells is associated with the activation of the Wnt/β-catenin pathway. EMT phenotypes can be reduced and the radiosensitivity of esophageal cancer cells can be enhanced by inhibiting the Wnt/β-catenin pathway with FH535 treatment.

## Background

Esophageal cancer is the eighth most frequently diagnosed cancer and the sixth most common cause of cancer-related death in the world [1,2]. Radiation therapy (RT) plays a crucial role in the treatment of esophageal cancer [3]. Although complex multidisciplinary treatment, especially the concurrent chemoradiotherapy, has recently become a common practice, the rate of local recurrence and distant metastasis remains high [4,5]. Acquired radioresistance during radiotherapy has been considered as one of the most important reasons for treatment failure [6].

Epithelial to mesenchymal transition (EMT) is a fundamental biological process during which epithelial cells lose their polarity and change to a mesenchymal phenotype [7]. The roles of EMT in tumor invasion, metastasis, chemotherapy resistance and cancer stem cells have been investigated intensively [8-11]. Emerging evidence suggests that EMT plays an important role in cancer radiation resistance [12,13]. Many radioresistant cancer cells demonstrated epithelial-mesenchymal transition (EMT), which was believed to link to adaptation to hypoxia, enhanced DNA repair ability and activated growth factor pathways [14-17]. However, the role of EMT in radioresistant acquirement during the radiotherapy of esophageal cancer remains unclear.

Previously, we found that the Wnt/β-catenin pathway may be the most important signaling pathway involved in this process based on cDNA microarray analysis of the KYSE-150R cell line [18,19]. In this study, we tested the hypothesis that EMT was involved in the acquired radioresistance of esophageal cancer cells via Wnt/β-catenin pathway. Our results indicated that KYSE-150R cell line displayed obvious radiation resistance and demonstrated an EMT-like phenotype together with the abnormal activation of Wnt/β-catenin signal pathway compared with parental cell line. Using the β-Catenin/Tcf inhibitor FH535, the expression of Wnt/β-catenin pathway proteins as well as EMT phenotypes were reduced, and at the same time, radiosensitivity was greatly increased.

## Methods

### Cell culture and reagents

Human esophageal squamous cancer cell line KYSE-150 were purchased from the American Type Culture Collection (Manassas, VA). Radioresistant cell line KYSE-150R was previously established in our department with gradient dose irradiation [18,19]. Both KYSE-150 and KYSE-150R were cultured in RPMI-1640 (Gibco, Life Technologies Inc., Grand Island, NY) with 100 unit/ml of penicillin, 100 mg/ml of streptomycin, and 10% fetal bovine serum at 37°C in a humidified incubator containing 5% CO_2_. The cell lines were sub-cultured every 2 to 3 days following digestion at room temperature with 0.5 ml trypsin/EDTA per well (Sigma-Aldrich Ltd, UK). The viability was reported as the percentage of the viable cells number to the total cells number. There was an average viability of over 95% determined by Trypan Blue staining. FH535 was purchased from Calbiochem (merck), and dissolved in DMSO at a concentration of 10 mM.

### Western blotting

Total proteins were extracted from the two cells groups (KYSE-150 and KYSE-150R) in the exponential growth phase using the cell lysis buffer, which containing 20 mM Tris (pH 7.5), 150 mM NaCl, 1% Triton X-100, 1 mM EGTA, 1 mM EDTA, 1 mM PMSF, and protease inhibitor cocktail (Merck Millipore, Darmstadt, Germany). Nuclear and cytoplasmic proteins were isolated using Nuclear and Cytoplasmic Protein Extraction Kit (Sangon Biotech, Shanghai, China). Protein content was measured using the Bradford protein assay. Samples were denatured at 100°C for 10 minutes. Thirty microgram proteins were separated by 8% or 10% SDS polyacrylamide gels and transferred to nitrocellulose membrane (0.45 μm; Bio-Rad). Incubation with 5% nonfat dry milk in TBS-Tween 20 (TBST) for 2 h at room temperature was conducted to block nonspecific binding sites. Subsequently, membranes were incubated with rabbit antibodies against β-catenin (H-102) (1:500) (ab2365, Cambridge, Mass., USA), Beta-Catenin Phospho (pS33/S37) (1:1,000) (9561 s, Cell Signaling Technology Danvers, USA), GSK3β (1:1,000) (1561–1, Epitomics, Inc., Burlingame, Calif., USA). P-GSK3β (1:1,000) (2435–1, Epitomics, Inc., Burlingame, Calif., USA)., Slug (1:1,000) (T0587, Epitomics, Inc., Burlingame, Calif., USA).E-Cadherin (1:1,000) (ab53033, Cambridge, Mass., USA), Vimentin (R28) (1:1,000) (#3932, Cell Signaling Technology Danvers, USA) and Cyclin D1 (1:1,000) (#2922, Cell Signaling Technology Danvers, USA) overnight at 4°C. Incubation with mouse antibodies against β-actin (1:10,000) (ab8226, Cambridge, Mass., USA) and GAPDH (1:4,000) (sc-32233, Santa Cruz, Calif., USA) were conducted for normalization control. After washing, membranes were again incubated with HRP-conjugated secondary antibodies (anti-mouse and anti-rabbit, 1:5,000) for 1 hour at room temperature. Membranes were then treated with enhanced chemiluminescence (ECL, Pierce, Thermo, USA) after washing. Proteins were visualized with ECL and Kodak film exposed for one minute without light. The relative band intensity was determined by Gel-Pro Analyzer 3.1 software (Media Cybernetics).

### Quantitative real-time RT-PCR analysis

Total RNA was extracted from the two cell groups (KYSE-150 and KYSE-150R) using Trizol Reagent (Invitrogen) according to the manufacture’s protocol and reversely transcribed into cDNA using Revert Aid First Strand cDNA Synthesis Kit (Fermentas, Thermo Scientific, USA). SYBR Green (ABI) PCR was performed in triplicate using the ABI PRISM 7300 Sequence Detection System. All samples were normalized to the signal generated from β-actin and GAPDH (Sangon Biotech, Shanghai, China). Primer sequences of Wnt pathway and EMT associated genes were presented in Table 1 Data was shown as fold change (2^−ΔΔCt^) and analyzed initially using Opticon Monitor Analysis Software V2.02 (MJ Research, Waltham, MA, USA). Triplicates were run for each sample in three independent experiments.

**Table 1:** **Primer sequences of Wnt pathway and EMT associated genes**

**Target gene**	**Forward primer (5′-3′)**	**Reverse primer (5′-3)**
β-actin	AAAGACCTGTACGCCAACAC	GTCATACTCCTGCTTGCTGAT
GAPDH	TCCCATCACCATCTTCCAGG	GATGACCCTTTTGGCTCCC
Cyclin D1	GCATCTACACCGACAACTCCA	GGCACAGAGGGCAACGA
E-cadherin	GAGAACGCATTGCCACATACA	GCGATGGCGGCATTGTA
Vimentin	CCAGATGCGTGAAATGGAA	TGGGTATCAACCAGAGGGAGT
Snail	CCCAATCGGAAGCCTAACTAC	AGCCTTTCCCACTGTCCTCA
Slug	TCCTGGTCAAGAAGCATTTCA	GGAGCAGCGGTAGTCCACA
Twist	CATGTCCGCGTCCCACTAG	CCACGCCCTGTTTCTTTGA
GSK3β	TGGCTACCATCCTTATTCCTCC	CCACGGTCTCCAGTATTAGCATC
FZD1	AAACAGCACAGGTTCTGCAAAA	TGGGCCCTCTCGTTCCTT
FZD2	TCCATCTGGTGGGTGATTCTG	CTCGTGGCCCCACTTCATT
FZD3	GCCTATAGCGAGTGTTCAAAACTCA	TGGAAACCTACTGCACTCCATATCT
FZD4	GCCCCAGAACGACCACAA	GGGCAAGGGAACCTCTTCAT
β-catenin	GCCAAGTGGGTGGTATAGAGG	GCGGGACAAAGGGCAAGA

### Immunofluorescence staining and confocal microscopy

KYRE-150R Cells were grown on covered slips and treated with 20 μM FH535 (purchased from Calbiochem and dissolved in DMSO) for 24 h. After washing three times with PBS, cells were fixed in 4% paraformaldehyde (Solarbio, Beijing, China) for 20 min at room temperature. The fixed cells were permeabilized with PBS containing 0.3% Triton X-100 (Solarbio, Beijing, China) for 20 min. Then, the cells were soaked in 10% normal goat serum (Solarbio, Beijing, China) with PBS for 30 min. The cells were then incubated with various primary antibodies (β-catenin, E-cadherin, Vimentin,1:200 dilutions) overnight at 4°C. After rinsing in PBS, the cells were then incubated with Alexa Flura® 488 conjugated, goat anti-rabbit IgG (H + L) (Thermo Scientific, Waltham, USA) at 1: 1000 dilution for 1 hr at 37°C at a dark condition. The nuclei were visualized by 1 μg/ml DAPI (Beyotime, Shanghai, China) and incubate for 5 min at room temperature. Images were obtained using a DM4000B Automated Upright Microscope System (Leica, Wetzlar, Germany).

### Cell viability assay

KYSE-150R cells, 1x10E3 cells/well, were seeded in a 96-well plate. After an overnight incubation, the cells were subcultured in three groups including: FH535 treated in different concentrations (5 μM, 10 μM, 15 μM, 20 μM), DMSO treated with the same volume as FH535 for 24 hours, and irradiation treated with a dose of 8 Gy high energy X-ray from a linear accelerator (Vairan 2300C/D, Salt Lake, USA). Cell counting kit-8 (CCK-8) assays (Dojindo) were performed following the protocol of the kits. Results were measured according to the absorbance at 450 nm using a microplate reader (Thermo Scientific Varioskan Flash, USA).

### Clonogenic assay

KYSE-150R cells, 1 × 10^3^ cells/well, in the exponential growth period were seeded in a 6-well plate for 24 h. FH535 was added with a concentrations of 20 μM for the following 24 hours. Then, the radioresistant KYSE-150R cells were irradiated by doses of 0, 2, 4, 6, 8, or 10 Gy with X-ray from a linear accelerator (Vairan 2300C/D, Salt Lake, USA) at an average dose rate of 100 cGy/min. Immediately following the irradiation, the cells were incubated for 10 days at 37°C in a 5% CO_2_ environment to allow the colony formation and then fixed with pure ethanol. Visible colonies consisting of at least 50 cells were stained with 0.5% crystal violet (Sigma, Germany) and counted. The surviving fraction (SF) were estimated.

### Comet assay

Neutral comet assay method was used to detect the DNA fragmentation associated with apoptosis. KYSE-150R cells were seeded at 2 × 10^5^ cells/well into 6-well plates and incubated overnight. From the three cell samples, two samples were then treated with 20 μM FH535 and DMSO for 24 hours, respectively. The other KYSE-150R sample were treated with 8 Gy irradiation. The Cells were collected and suspended in PBS with a concentration of 5 × 10^5^ cells/mL. To prepare the slide, the cell suspension (10 μl) was mixed with 90 μl of 0.75% low temperature melting agarose (LMA) in PBS at 37°C and pipetted onto fully frosted slides, which precoated with a layer of 100 μl 1% normal temperature melting agarose (NMA) in PBS. After gelling for 10 minutes at 4°C, the coverslip was gently removed. All the processes were conducted at a dark condition. The slides were placed in precooled lysis solution (2 M NaCl, 30 mM Na_2_EDTA, 10 mM Tris, 1% N-Lauroylsarcosine Sodium Salt, pH 8.2-8.5, supplemented with 10% DMSO and 1% Triton X-100) at 4 °C for 2 hours, and then placed in TBE (0.4 mM Na2EDTA, 18 mM Tris, 18 mM boronic acid, pH 8.2-8.5) at 4°C for 30 minutes twice. Electrophoresis was performed for 20 minutes at 25 V (10 mA) in TBE solution after unwinding. Slides were stained with propidium iodide (PI) (5 ug/ml) for 10 minutes and covered with a coverslip. Microscopic analysis were conducted using a fluorescence microscope (Leica DM2500, Germany) equipped with a CCD camera. Non-overlapping cells were captured at 400 magnification. At least 100 cells per slide were analyzed using the Comet Assay Software Project (CASP). A measurement of olive tail moment (OTM) was used to quantify the extent of DNA damage.

### Statistical analysis

The results were described as mean plus standard deviation (SD) for triplicate measurements. Statistically significant differences between groups were estimated by Student’s *t* test using SPSS (13.0). P < 0.05 was considered statistically significance.

## Results

### Acquisition of EMT phenotype in radioresistant esophageal cancer cells

**Figure 1:**
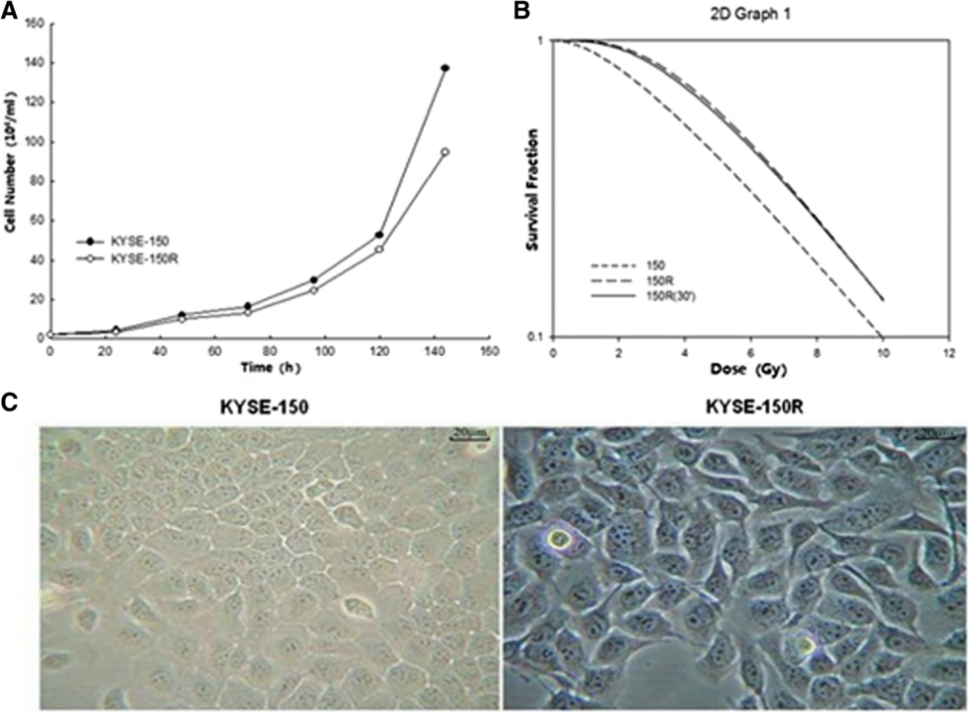
**The cell line KYSE-150R displayed significant radioresistance compared to the parental cell line KYSE-150. (A)** the growth curve of KYSE-150R cells and the parental cells. **(B)** Clonogenic survival assays of KYSE-150R group and control group. Surviving fractions were calculated by the number of colonies divided by the number of seeded cells multiplied by plating efficiency. **(C)** The KYSE-150R cells were morphologically distinct from the parental cell lines.

KYSE-150R cell line displayed significant radioresistance compared with the parental cell line (KYSE-150), with a reduced growth rate and significantly increased clonogenic survival (Figure 1A; B). The radioresistant phenotype was maintained for at least 3 months (or 30 passages) after the radiation. KYSE-150R cells were morphologically different from the parental cell lines (Figure 1C). The radioresistant cells demonstrated an irregular, elongated fibroblastoid morphology with a loss of cell-cell adhesion, while the parental cell lines maintained uniform cobblestone morphology with adhesions and tight junctions. These changes in phenotype suggested that KYSE-150R cells may have undergone the conventional EMT as reported previously.

**Figure 2:**
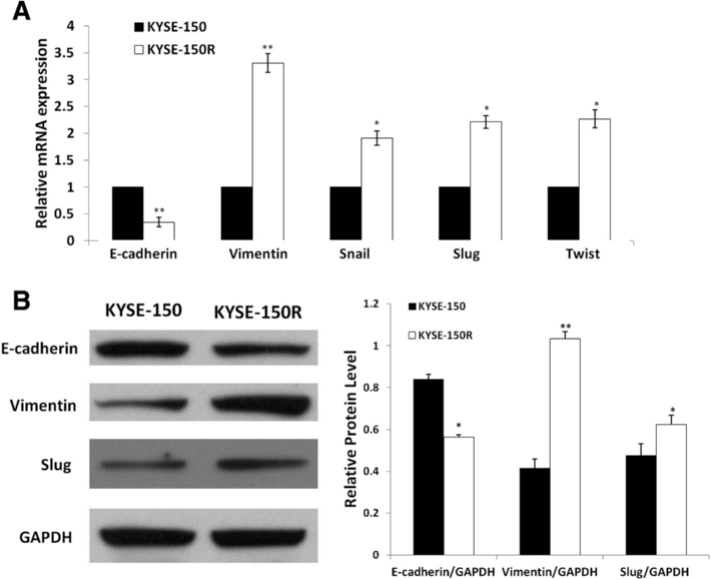
**EMT phenotype in radioresistant esophageal cancer cells.** Reduced expression of E-cadherin and increased expression of Vimentin, Slug, Twist and Snail were found in KYSE-150R cells. **(A)** EMT makers genes expression determined by reverse transcription polymerase chain reaction (RT-PCR). All samples were normalized to the signal generated from housekeeping gene (GAPDH). **(B)** Western blotting assessed E-cadherin, Vimentin and Slug in KYSE-150R group and control group. GAPDH was used as a loading control.

To determine whether this morphological transformation represents EMT, we analyzed the expression of EMT phenotype markers by RT-PCR. As shown in (Figure 2A), the expression of E-cadherin (CDH1) was decreased and the expressions of Vimentin (VIM), Snail, Slug and Twist were increased in KYSE-150R cells compared with those in KYSE-150 cells. Similar results were observed at the protein level by western blotting as shown in Figure 2B. These results indicated that the radioresistant esophageal cancer cells have indeed transformed to a mesenchymal state.

### Activation of Wnt pathway in KYSE-150R cell

**Figure 3:**
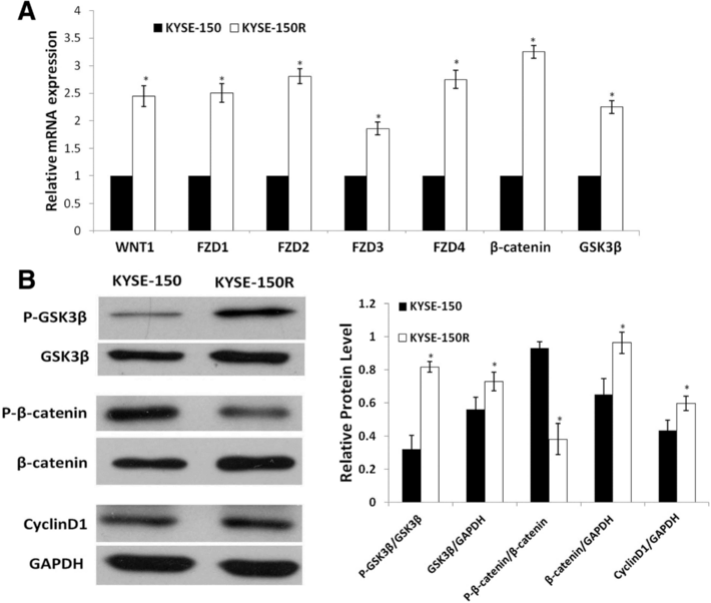
**Activation of Wnt pathway constitutively in radioresistant esophageal cancer cell. (A)** mRNA expression of Wnt pathway protein was measured using RT-PCR. Graph represents the mRNA fold-regulation values of radioresistant KYSE-150R cells relative to parental cells, normalized against housekeeping gene (GAPDH) with standard deviation of triplicate experiments represented by error bars. **(B)** Protein level confirmation of activated Wnt signaling markers using western blot. GAPDH was the loading control. Data are representative of at least 3 independent experiments.

Our preliminary whole genome microarray analysis implicated the Wnt signaling genes were highly significant (data not shown). We therefore quantified the mRNA expression of Wnt/β-catenin signaling components in both the parental and radioresistant cells by RT-QPCR. The expressions of Wnt ligands Wnt1, Wnt receptors frizzled 1–4 (FZD1-4), the intracellular signal transducers GSK3β and CTNNB1, as well as targeted genes Cyclin D1 were all significantly up-regulated in the KYSE-150R cells (Figure 3A).

An increase in phosphorylation of glycogen synthase kinase 3β (GSK3β) along with attenuated GSK3β activity in radioresistant cells was observed compared with their parental cells (Figure 3B). The phosphorylations of Thr 41 and Ser 45 in β-catenin, the important regulatory sites for proteasomal degradation of the transcription factors, decreased in the KYSE-150R cells. The protein levels of cyclin D1 were significantly increased in KYSE-150R cells (Figure 3B).

### Wnt/β-catenin signaling pathway inactivation by FH535

**Figure 4:**
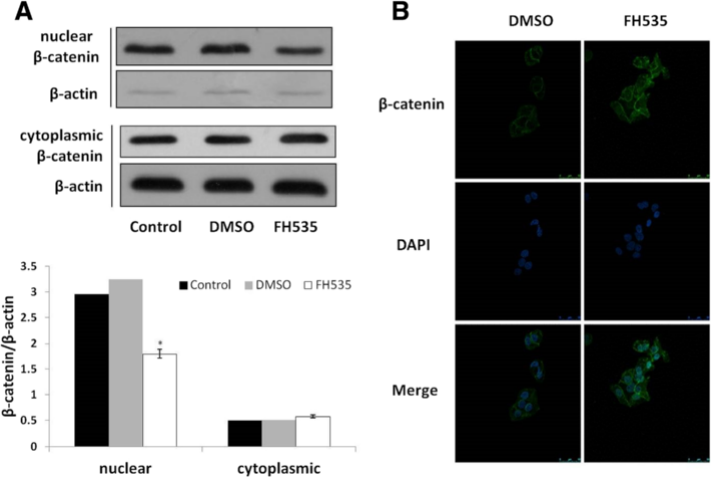
**Repression of nuclear translocation of β-catenin with FH535. (A)** Western blotting analysis determined Nuclear and cytoplasmic expression of β-catenin in the radioresistant KYSE-150R cells treated with or without FH535. β-actin was the loading control. Data are representative of at least 3 independent experiments. **(B)** Expression of the β-catenin detected by immunofluorescence analysis. Nuclei were counterstained with DAPI (blue). The images were zoomed in 400×.

To further investigate the role of the Wnt/β-catenin signaling pathway in EMT, a reversible inhibitor of the Wnt pathway, FH535, was used. The expressions of nuclear and cytoplasmic β-catenin were analyzed following FH535 treatment. Western blot results demonstrated that nuclear β-catenin decreased, while cytoplasmicβ-catenin slightly increased (Figure 4A) after FH535 treatment. Average optical density of the β-catenin was analyzed using Image-pro Plus. The increased expression of β-catenin in radioresistant KYSE-150R cells treated with FH535 was 0.044 ± 0.0002, then the average optical density of non-FH535 treated was 0.031 ± 0.0010 (P = 0.004) (Figure 4B).

### EMT suppression in KYSE-150R cell line by FH535

**Figure 5:**
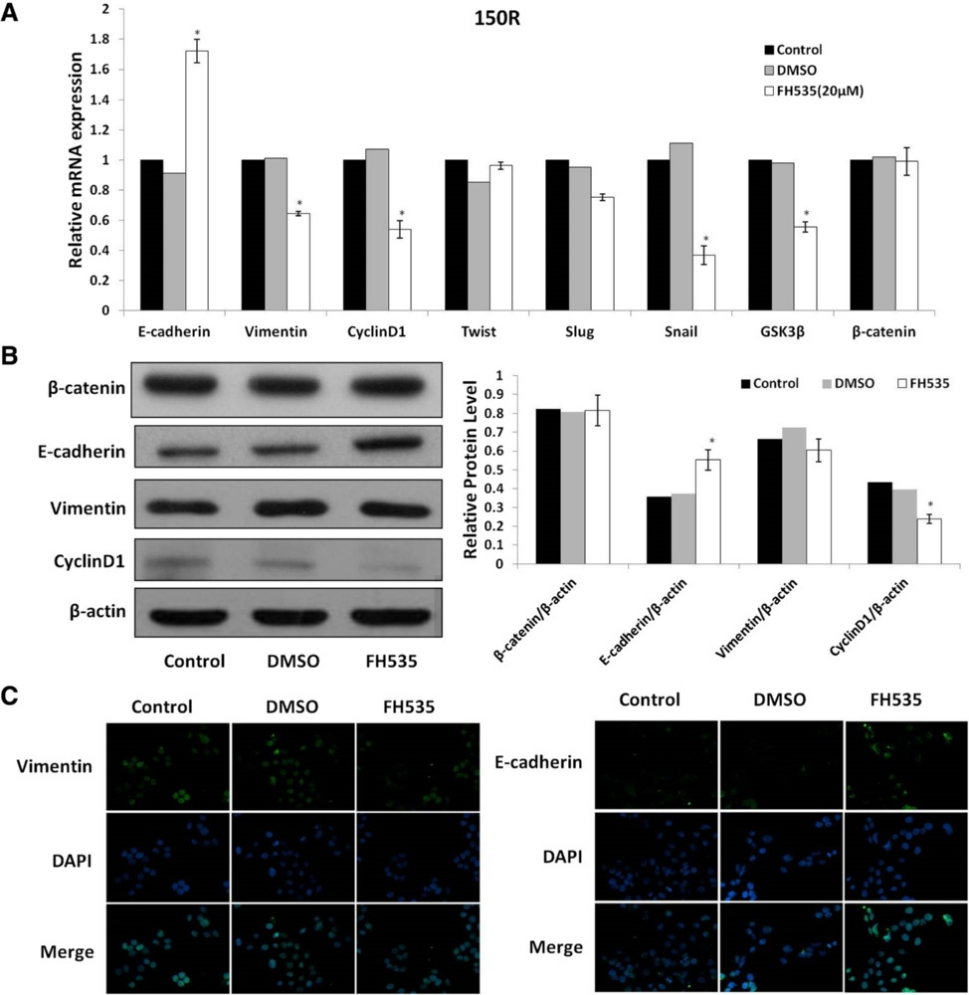
**EMT suppress in KYSE-150R cell line with FH535. (A)** The effect of FH535 on EMT target genes expression determined by RT-PCR. All samples were normalized to the signal generated from housekeeping gene (β-actin). **(B)** Western blot analysis of EMT protein levels treated with FH535 for 24 h, GAPDH was used as normalization control. **(C)** Immunofluorescence analysis showed protein levels of E-cadherin and Vimentin. Nuclei were counterstained with DAPI (blue). The images were zoomed in 400×.

Next, we sought to test whether FH535 could affect the EMT markers in the treated KYSE-150R cell line. A statistically significant increase of E-cadherin mRNA and decreases of Vimentin (VIM)、Snail and GSK3β mRNAs after FH535 treatment were observed in the KYSE-150R cells (Figure 5A). Consistently, Western blot and immunofluorescence analysis results showed significantly increase of protein levels of E-cadherin in the KYSE-150R cells (Figure 5B). The increased expression of E-cadherin in radioresistant KYSE-150R cells treated with FH535 was 0.081 ± 0.0028, and the average optical density of group without FH535 treatment was 0.056 ± 0.0025 (*p* = 0.01). However, no significant difference in the expression of Vimentin was observed between FH535 treated groups and controls in both western blot and immunofluorescence analysis. The expression of Vimentin in radioresistant KYSE-150R cells treated with FH535 was 0.042 ± 0.0016, and the average optical density of group without FH535 treatment was 0.040 ± 0.0005 (*p* = 0.222). Interestingly, the Wnt pathway target gene Cyclin D1 was down-regulated specifically in the FH535 treatment group in both mRNA and protein level, indicating the inhibition of the Wnt/β-catenin pathway.

### FH535 acts as a potential radiosensitizer in KYSE-150R cells

**Figure 6:**
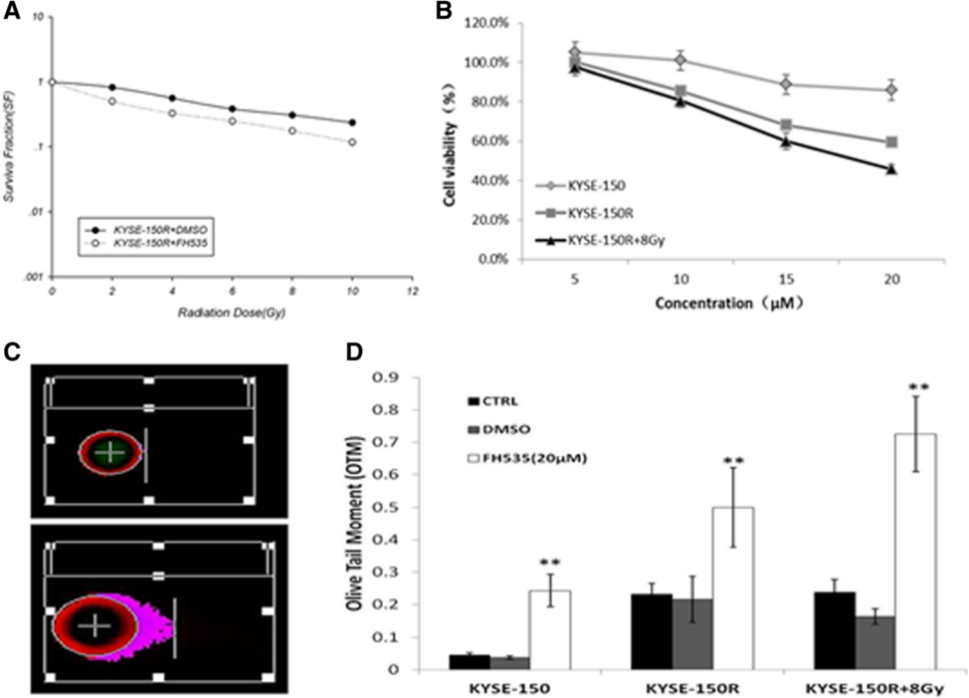
**FH535 acts as a potential radiosensitizer in KYSE-150R cells. (A)** Clonogenic survival in FH535 (20 μM) treated KYSE-150R cells after irradiation. The data points show mean survival fraction from 3 individual experiments. **(B)** CCK8 proliferation assays were performed on the KYSE-150R cells treated with increasing doses of FH535 (0.0-20 μM) over 24 hours. Graph represents the average cell proliferation in percentage of 6 replicates with standard deviation represented by error bars. **(C)** Comet assay of DNA damage response. The cells were cultured for the given periods and then subject to agarose gel electrophoresis. The photographs show the momentum of the nuclear DNA movement. **(D)** Summary data of momentum of cellular DNA. Data shown from three independent experiments. *P < 0.05.

We tested whether FH535 could potentially act as a radiosensitizer for the KYSE-150R cells by CCK8 analysis and a colony forming assay. The proliferation rates of the KYSE-150R cells was reduced in a dose-dependent manner with FH535 treatment, as assessed by the CCK8 assay. The combined treatment of FH535 and irradiation (8 Gy) effectively suppressed the proliferation rates compared with FH535 treatment alone (Figure 6A). The results of colony forming assays showed that FH535 treatment significantly enhanced the radiotherapy effect in the KYSE-150R cells (Figure 6B). The characteristics of cell survival curves were presented in Table 2. The SF_2_ (survival fraction at 2 Gy) in the KYSE-150R cells treated with FH535 and DMSO were (0.50 ± 0.05) and (0.80 ± 0.02), respectively (p < 0.05). The neutral comet assays was applied to quantify the double strand break (DSB) levels. The momentum was significantly increased in the FH535 treatment group (Figure 6C and D), implicating that the DNA DSB repairing was impaired by FH535 in the KYSE-150R cells.

**Table 2:** **The radiobiology parameters of these two different groups were compared by cell survival curve**

	**Do**	**Dq**	**SF**_**2**_
DMSO	5.43	2.00	0.80
FH535	6.80	1.25	0.50

## Discussion

In this study, the roles of EMT and Wnt/β-catenin signaling pathway in radioresistance were investigated in a radioresistant esophageal cancer cell line (KYSE-150R) that showed a decrease in growth rate and significantly increase in clonogenic survival compared to its parental cells after irradiation. Phenotypic changes similar to EMT and activation of the Wnt/β-catenin signaling pathway were observed in KYSE-150R cells compared to its parental cells.

EMT-like phenotype and its close relationship with radioresistance have been investigated in breast carcinoma and lung carcinoma cells [15,16,20]. It has also been reported that radioresistance associated with EMT and enhanced cancer stem cell phenotypes in prostate cancer [21]. Consistently, in the present study, EMT-like phenotype was also discovered in the radioresistant esophageal cancer cells (KYSE-150R) with irregular shape and a loss of cell-cell adhesion. Further RT-PCR results with the significant decrease of E-cadherin and increase of snail and twist expressions confirmed the presence of EMT.

Wnt family proteins consist of two subfamilies based on downstream intracellular signaling. The canonical Wnt pathway stabilizes β-catenin and activates target genes via TCF/Lef transcription factors. Wu et al. demonstrated that the increase of Wnt3 promoted a partial epithelial-to-mesenchymal transition presenting with increased N-cadherin, Twist, Slug and decreased E-cadherin [22]. Knockdown of Wnt3 by siRNA decreased the EMT markers, increased E-cadherin expression, and inhibited the cell invasiveness [22]. Zhao et al. discovered that overexpression of HIF-1α, which stimulated the invasion potency of human prostate carcinoma cells through EMT and Wnt/β-catenin signaling pathway, might be a necessary endogenous signal [23]. In this study, significantly up-regulated expressions of Wnt1, FZD1-4, GSK3β, CTNNB1 and Cyclin D1, as well as increase in phosphorylation of GSK3β and decrease of β-catenin were observed in the radioresistant esophageal cancer KYSE-150R cells, implicating the activation of the Wnt/β-catenin signaling pathway. Similarly, several studies suggested that aberrant activation of canonical Wnt/β-catenin signaling after long-term exposure to fractionated irradiation was associated with the development of radioresistance in many human cancers [24,25].

In the present study, a reversible Wnt pathway inhibitor, FH535, was used to further investigate the relationship between the activation of the Wnt/β-catenin signaling pathway and EMT phenotype change in esophageal cancer radioresistance. Previous studies have shown that FH535 is a synthetic inhibitor of the canonical Wnt/β-catenin signaling pathway [26]. It significantly inhibits the growth of the cells of breast cancer, colon cancer, lung cancer, and hepatocellular carcinoma, but not normal fibroblasts [27]. Consistently, the expression of Wnt/β-catenin pathway protein as well as EMT phenotypes in KYSE-150R cells were reduced with the treatment of FH535 in this study. At the same time, radiosensitivity of KYSE-150R cells was greatly increased. These results indicated that radioresistance in KYSE-150R cells can be reverted via EMT reversal and down-regulation of Wnt/β-catenin pathway with FH535 treatment.

Recent studies have shown that several small molecular inhibitors can enhance radioresistivity by interfering the Wnt/β-catenin pathway, such as GDK-100017. GDK-100017 is a small inhibitor that can reduce TCF/LEF dependent transcriptional activity and possesses potential anti-cancer activity against human non-small cell lung cancer (NSCLC) by inhibiting the Wnt/β-catenin pathway to enhance the radiosensitivity [28]. Interestingly, in our study, FH535 not only acted as a potential radiosensitizer by blocking the Wnt/β-catenin pathway, but also effectively suppressed EMT. This mechanism was also observed in NSCLC in which FH535 could reverse the phenotype of TGF-b1-induced EMT, which is a potential treatment target for EMT reversal [29].

## Conclusion

This study demonstrated that esophageal cancer radioresistance is associated with EMT via the activation of Wnt/β-catenin signaling pathway. FH535 could reduce EMT phenotypes and increase esophageal cancer radiosensitivity. Inhibiting the Wnt/β-catenin pathway maybe a promising strategy to overcome radioresistance in the treatment of esophageal cancer radioresistance.
